# MEBO versus topical Diltiazem versus a combination of both ointments in the treatment of acute anal fissure: a randomized clinical trial protocol

**DOI:** 10.1186/s12906-021-03227-z

**Published:** 2021-02-24

**Authors:** Mohamad Hadi El Charif, Samer Doughan, Rawya Kredly, Sara Kassas, Rayan Azab, Eman Sbaity

**Affiliations:** 1 Department of Surgery, American University of Beirut Medical Center, Riad El-Solh, Beirut, Lebanon; 2 Office of Medical Affairs, Gulf Pharmaceutical Industries – Julphar, United Arab Emirates, Beirut-Lebanon Offices, Beirut, Lebanon; 3 Faculty of Health Sciences, American University of Beirut, Beirut, Lebanon; 4 Gulf Pharmaceutical Industries – Julphar, United Arab Emirates, Beirut-Lebanon Offices, Beirut, Lebanon

**Keywords:** Anal fissure, Mebo ointment, Herbal, Natural, Diltiazem ointment, Randomized clinical trial

## Abstract

**Background:**

Anal fissure is a common complication of the anorectal region and one of the most reported causes of anal pain. Acute anal fissure can be cured by surgery or medical treatment. There is an increase in the use of topical therapy for the treatment of anal fissures. A common topical drug used is Diltiazem (DTZ), a calcium-channel blocker, which relaxes the anal sphincter and thus promotes healing of the anal fissure. Moist exposed burn ointment (MEBO) is an ointment that is effective for the treatment of burns and wound healing and is becoming popular in the treatment of anal fissures.

**Methods:**

This is a 1:1:1 randomized, controlled, parallel design, with endpoint measures of change in pain score, wound healing, defecation strain score and patient’s global impression of improvement. The study will be conducted at AUBMC over a 10-week period. Patients will be randomized to three treatment arms: MEBO, Diltiazem, and a combination of MEBO and Diltiazem ointments.

**Discussion:**

The results of this study will allow physicians to assess the efficacy and safety of MEBO in the treatment of acute anal fissure, and also in comparison to Diltiazem. This trial will generate evidence-based conclusions regarding the use of a herbal/natural-based product (MEBO ointment) for the treatment of anal fissures.

**Trial registration:**

ClinicalTrials.gov Identifier NCT04153032. Clinical Trial Registration Date: 06-NOVEMBER-2019.

**Supplementary Information:**

The online version contains supplementary material available at 10.1186/s12906-021-03227-z.

## World Health Organization trial registration data set

1. Primary Registry and Trial Identifying Number: NIH Clinicaltrials.gov; NCT04153032
2. Date of Registration in Primary Registry: 06-NOVEMBER-2019
3. Secondary Identifying Numbers: BIO-2018-0547
4. Source(s) of Monetary or Material Support: Gulf Pharmaceutical Industries – JULPHAR
5. Primary Sponsor: Eman Sbaity, MD
6. Secondary Sponsor(s): Not Applicable
7. Contact for Public Queries: Mohamad Hadi El Charif, MD (Riad el Solh 1107 2020, Beirut – Lebanon; 00961–3-771,917; AUBMC Medical Center Phase 1 - Department of Surgery - 4th Floor)
8. Contact for Scientific Queries: es25@aub.edu.lb or me209@aub.edu.lb (Riad el Solh 1107 2020, Beirut – Lebanon; 00961–3-771,917; AUBMC Medical Center Phase 1 - Department of Surgery - 4th Floor)
9. Public Title: Comparative Evaluation of MEBO Ointment and Topical Diltiazem Ointment in the Treatment of Acute Anal Fissure
10. Scientific Title: MEBO versus topical Diltiazem versus a combination of both ointments in the treatment of acute anal fissure: a randomized clinical trial protocol (MEBO)
11. Countries of Recruitment: Lebanon
12. Health Condition(s) or Problem(s) Studied: Acute Anal Fissures
13. Intervention(s): *Listed in the submitted protocol*
14. Key Inclusion and Exclusion Criteria: *Listed in the submitted protocol*
15. Study Type: Interventional; Randomized; Single-Blinded; Parallel; Comparative; Phase 2
16. Date of First Enrollment: None yet
17. Sample Size: Intended number is 183
18. Recruitment Status: Recruiting
19. Primary Outcome: *Listed in the submitted protocol*
20. Key Secondary Outcomes: *Listed in the submitted protocol*
21. Ethics Review: Approved on September 2018 by IRB at AUBMC (Ethics committeee contact: Mrs. Karine Ismail ki09@aub.edu.lb)
22. Completion Date: Estimated by June 2021
23. Summary Results: Not Applicable Yet
24. IPD Sharing Statement: There is a plan to share IPD upon reasonable request during the trial period and for 3 years post-trial completion. Beyond that the IPD will be available through data archive registry.

## Background

Anal fissure is one of the most common anorectal complications encountered in practice [1] that represents a common cause of intense anal pain [2]. Anal fissure is linked to reduced quality of life and loss of working hours [2]. It occurs in all age groups, and is equally prevalent in men and women [3]. Anal fissure is a longitudinal tear at the epithelial lining of the anus [4]. During defecation, stretching of the lesion causes severe pain, that persists hours after defecation and many times is accompanied by bleeding [5]. A minority of patients also experience additional symptoms such as swelling, prolapse, pruritus, and discharge [6].

The majority of anal fissures are acute and resolve spontaneously [7] or within 6–8 weeks of conservative treatment [8]. Anal fissures with symptoms that persist longer are classified as chronic anal fissure [1]. The possible mechanism for acute anal fissure is straining during defecation, especially constipation [1] leading to trauma [9, 10].

The primary method for diagnosing anal fissures is by clinical physical examination nevertheless, some other diagnostic modalities have become a topic of interest for more accurate descriptive diagnosis and classification of anal fissures. Anorectal manometry is not routinely used in the diagnosis and assessment of anal fissures, acute or chronic. However, historically it was found that patients with anal fissures have the highest squeeze and maximal basal pressures on manometric measurements [11, 12]. The value of anorectal manometry resides in the pre-operative assessment of invasive and non-invasive procedures such as sphincterotomy and anal dilatation [12, 13]. The study by Gil et al. shed a light on a new utility for manometry in anal fissures which is stratifying patients in light of allocated conservative treatment success [14]. Even more, Bove et al. describes how anal pressure variances can guide the healthcare provider to confidently recommend one choice of treatment or another [15].

Adequately managing anal fissures is pivotal to the improvement of the quality of life and daily functioning of patients. This is reflected in the findings of studies assessing the health-related quality of life, in patients complaining of anal fissures, before and after medical management [8, 16, 17]. In one study, the authors found that anal fissures had a negative effect on the pain scale as well as the irritability scale [8]. In another study, it was noted that the longer the symptoms lasted, the poorer the rates of healing and pain were and subsequently management with conservative medical treatment caused a rapid relief of symptoms which encouraged better healing and a reduced pain score [16].

Acute anal fissure is often treated through the ingestion of bulking agents such as fibers, sitz baths and, with or without the application of local ointments [18]. It is also suggested that the addition of topical ointments accelerates the healing process and prevents the progression to chronic anal fissure [19]. Topical ointments are increasingly being used to treat chronic anal fissure due to the cost-effectiveness and to avoid postsurgical complications [20]. These topical anesthetics include nitrates and calcium channel blockers (CCBs) that heal the fissure through relaxation of the anal sphincter [7, 21].

Recent studies suggest that addressing the microbial colonization of the anorectal region in patients complaining of anal fissures could yield better treatment outcomes [22]. Moreover, trials of topical and oral antibiotics in addition to the usual indicated management of anal fissures showed significant improvement in terms of wound healing and pain score [23, 24]. The review by Garg et al. stresses on the importance of absolute avoidance of constipation in order to achieve the best rate of fissure resolution [23].

Several studies were conducted on DTZ, a CCB, for the treatment of chronic anal fissure [1, 7] . A systematic review that compared DTZ to other drugs in the treatment of chronic anal fissure found that oral and topical applications of DTZ significantly reduced anal pressure and that compared to nitrate, DTZ had minimal side effects [7]. Another systematic review on DTZ in the treatment of chronic anal fissure suggested that the application of DTZ, twice or three times per day with daily doses between 16 and 28 mg is an effective therapy for chronic anal fissure [1]. In regard to the treatment of acute anal fissure, there is a dearth of studies on the use of DTZ for acute anal fissure healing. However, studies were conducted on another type of CCB, nifedipine, as a topical medical therapy for acute anal fissure [19, 25].

Moist exposed burn ointment (MEBO) is a topical Chinese medicine composed of sesame oil and herbal plants [26] that is clinically used for the treatment of burn wounds [27]. A study that compared the efficacy of MEBO to a standard therapy in the treatment of partial-thickness facial burns reported comparable rates of wound healing. The study also reported that MEBO allowed easier assessment of healing progression, easier application, and was cost-effective [28]. On the other hand, a randomized controlled clinical trial revealed that MEBO effectively improved pressure ulcer healing compared to placebo [26]. The possible mechanisms by which MEBO promotes wound healing include increasing neovascularization and fibroblast cells in granulation tissue, and enhancing proliferation of the vascular endothelial cells [26]. In the Chinese literature, studies that were conducted on MEBO in the treatment of anal fissures found that MEBO is effective in healing anal fissure [29, 30].

MEBO is not available in Europe and USA. It has proven effectiveness in burn management and wound healing, and is widely used in Asia and Middle East, including Lebanon. Currently there is a phase II trial registered on clinicaltrial.gov to study effect of MEBO of burns as compared to standard of care. This is a step in the process of FDA approval of MEBO. Since it is not yet approved for use in USA and Europe, it is not included in clinical practice guidelines in both regions.

No study to date has compared topical DTZ to MEBO ointment in the treatment of anal fissure. Moreover, in the absence of data comparing MEBO to other topical agents, and since there is no single standard of care, we propose to conduct a comparative randomized clinical study. In this study, we will compare patients with acute anal fissure receiving MEBO Ointment 30 g (0.25% w/w beta-sitosterol) versus topical DTZ ointment 30 g (2%) versus a combination of MEBO and DTZ ointment.

### Rationale

Despite the significant medical arsenal available today, there are only a limited number of approved medications for the management of anal fissures. In addition, aside from the use of MEBO in Chinese and other Eastern cultures, we do not know of a natural product utilized to the latter end. In addition, no study to date has evaluated the use of MEBO for anal fissures nor compared topical DTZ to MEBO ointment in the treatment of anal fissure. In the present study, we aim to compare the efficacy and safety of DTZ to MEBO in the treatment of acute anal fissure.

#### Hypothesis

MEBO in combination with DTZ is more effective than DTZ or MEBO alone in the treatment of acute anal fissure.

## Methods/design

### Aims of the study

The main aim of this study is to evaluate the efficacy and adverse effects of MEBO Ointment and Topical DTZ ointment, when administered as single agents, or as a combination, in the treatment of acute anal fissure.

### Trial design

This trial is designed as a 1:1:1 randomized, controlled, parallel design. The primary endpoint is change in pain score from pre-treatment to 1 week from the start date of the study; secondary endpoints are change in pain score at 6 and 10 weeks and wound healing at 1, 6 and 10 weeks from the start date of the study, defecation strain score and patient’s global impression of improvement at 10 weeks.

### Study setting

We will conduct the study at the American University of Beirut Medical Center, which is an academic, tertiary referral center.

### Provision of interventional treatments

The funder of this clinical trial will be responsible for providing the new interventional ointment Mebo in the form of tubes of 0.25% W/W beta-sitosterol which is the exact same formula and package supplied to the pharmaceutical market. These tubes will be provided for the participants randomized to the Mebo and Combination arms for free.

The funder of this clinical trial is also responsible for providing or covering the cost for the Diltiazem ointment. The ointment form of Diltiazem is not available in the Lebanese pharmaceutical market and as such it has been and will remain to be formulated and dispensed by AUBMC’s pharmacy based on treating surgeons’ prescriptions. The DTZ ointment is provided in small plastic jar containers. These jars will be provided to the participants randomized to the DTZ and Combination arms for free.

### Eligibility criteria

#### Inclusion criteria

- Subjects must be 18 years and above.
- Subjects with 3 months (90 days) or less history of painful anal fissure (AF)*,* prior to screening, where AF-related pain-associated with, or following, defecation is experienced at least twice a week during the symptomatic phase, with pain scores at an average of ≥3 on an 11-point NRS (Numerical Rating Scale, range 0–10 where 0 = no pain and 10 = worst pain imaginable).
- Subjects with an average of ≥4 on an 11-point NRS during the screening phase for worst anal pain associated with, or following, defecation for the most recent 3 days on which the subject has defecated.
- Subjects with evidence of a radial fissure, with induration at the edges, seen on anal examination.
- Are willing to stop all other concomitant topical preparations applied perianally prior to commencing study treatment, and throughout the study. There will be a “washout” period of at least 2 weeks prior to commencing the study for subjects who were using other concomitant topical preparations applied perianally.
- Able to give consent

#### Exclusion criteria

- Subjects that:
- Are unwilling to be examined for AF.
- Who have undergone a Lateral sphincterotomy or anal stretch or other previous interventions involving the anal canal or perianal region
- Who have had sub-fissure injection of botulinum toxin within 6 months period or used glyceryl trinitrate (GTN) ointment for > 1 week in the 4 weeks prior to the screening visit
- Have AF associated with other conditions (drug-induced [e.g. nicorandil], trauma, HIV infection, fistula-in-ano, inflammatory bowel disease, perianal sepsis or malignancy).
- Have cardiovascular disease, inflammatory bowel disease, chronic fecal incontinence, history of radiation therapy to the pelvis or fixed anal stenosis/fibrosis, major psychiatric (including drug or alcohol abusers), or hematological illness
- Have a known hypersensitivity to DTZ or the ingredients of MEBO
- Are on medications prohibited by the protocol.
- Have taken experimental agents within 8 weeks or a period equivalent to 5 half-lives (t_1/2_) of the agents prior to screening
- Have a planned elective or other treatment requiring hospitalization, during the study, booked before entry into the study
  - Will be unavailable for the duration of the trial, likely to be noncompliant with the protocol, or who are felt to be unsuitable by the Investigator for any other reason
- Are taking oral therapy for anal fissure

### Concomitant medications

#### Allowed

- Concomitant laxatives and stool softeners will be permitted, as needed, during the entire study period (treatment and follow up) to ensure that constipation or passage of hard stools does not confound evaluation or improvement of the condition.
- Pain killers

#### Prohibited

- Any new medication for AF will not be permitted unless the Investigator deems “rescue” intervention necessary. The patient’s treating physician will allocate the patient to this intervention. Subjects eligible for the rescue intervention will be excluded from the trial.
- β-adrenoceptor antagonists
- CCBs

### Physician’s eligibility

Physicians who want to enroll their patients into the trial have to be familiar with the treatment of acute anal fissure. They will have to attend a presentation given by the study principal investigator explaining the details of the study.

### Interventions

The first group of patients will apply MEBO ointment 0.25% peri-anally 3 times daily for 6 weeks. The second group of patients will apply topical DTZ ointment 2% peri-anally 3 times daily for 6 weeks. The third group will apply a combination of MEBO (once per day in the evening) and DTZ ointment (twice per day, in the morning and afternoon) peri-anally 3 times daily for 6 weeks. The Diltiazem ointment is prepared by our AUBMC pharmacy for clinical use in treating anal fissures. So, we will use this same preparation for the research study. Sterile preparation and preservation conditions are as determined by the pharmacy. We will instruct the patient in clinic how to apply the ointment whether MEBO or Diltiazem, and the patient applies it himself. This is current clinical practice and it will be the same in the research setting.

Those who fail therapy (meaning they report no pain improvement after 2 weeks of treatment) from either the MEBO or the Diltiazem arm will be switched to the combination arm. If they also fail to improve after 2 weeks on the treatment arm, then they will be offered surgery. If the patient refuses to switch to the combination arm and wishes to proceed to surgery immediately, then the patient will be directed to surgery.

### Strategies to improve adherence to intervention protocol

The principal investigator (PI) will plan a lecture to address physicians who will be administering MEBO and DTZ for patients with acute anal fissure.

It is the responsibility of the PI/study team to ensure proper and timely assessment of outcome measures, including timely visits by the physician and scheduling of appointments at the outpatient department. Upon discharge from the clinic, the patients will be given a calendar as timeline chart on the dates of the remaining follow updates. The study team will call the patients 1 day before the appointments to ensure they will show up. Patients will also be called once per week from the start date of the study to ensure that they are adhering to the medication application, laxative or fiber supplements they were taking at baseline. Afterwards, they will be followed up 1 and 6 weeks from the start date of the study during which they will receive the treatment. Follow-up will be done 4 weeks after the treatment period.

We will give the patients detailed written instructions on how to apply the medication. The tubes of medication that are given to the patients will be weighed at baseline, 1st and 6th week to assess compliance with the advised amount of ointment.

### Relevant concomitant care and interventions during the trial

Patients will be instructed to adhere to medications, laxatives, or fiber supplements they were taking at baseline to relieve pain or constipation.

### Outcomes

#### Primary outcome measures

- Change in pain score, will be measured at baseline (i.e. start date of the study) and 1 week from the start date of the study. Pain is evaluated through the Numerical Rating Scale, range 0–10 where 0 = no pain and 10 = worst pain imaginable [31]. (see Appendix A).

#### Secondary outcome measures

- Change in pain score at 6 and 10 weeks from the start date of the study.
- Wound healing will be measured at 1,6 and 10 weeks from that start date of the study. Wound healing is assessed through physical examination: digital rectal examination is not done in case of painful anal fissure. Assessment for wound healing will be performed by the treating physician of the patient. The patient will be asked to strain to make the anal fissure more visible. If it is difficult to see on physical examination, digital rectal exam will be done carefully. The degree of healing will be reported as none, partial or complete.
- Defecation strain score. Level of strain during defecation is graded on a four-point scale: serious strain is scored as 3, moderate strain as 2, mild strain as 1, and defecation without strain as 0 [32]. It will be measured at 1 week, 6 weeks, and 10 weeks from that start date of the study.
- The patient’s global impression of improvement will be measured on a 7-point Likert scale. It will be measured at 1 week, 6 weeks, and 10 weeks from that start date of the study [33]. (see Appendix B).

#### Exploratory outcomes (safety evaluation)

- Incidence of treatment-related adverse events that include headache, itching, dizziness, and vital signs and sensitivity reactions will be evaluated. Adverse events will be measured at 1 week, 6 weeks, and 10 weeks from that start date of the study.

### Sample size

We will design the trial as a three-arms superiority trial to determine which treatment arm is better. Sample size is calculated accordingly. Assuming that the change in pain score difference between both treatments will be 0.5 points with a 1-point standard deviation on the NRS scale (5 for DTZ and 3 for combination arm DTZ and MEBO) and using an alpha of 0.05 and a power of 80%, we will need a sample size of 47 patients in each arm. After accounting for 30% loss to follow up, we will need to recruit 61 patients in each arm. Total number of patients to be recruited in the three arms is 183 patients.

## Discussion

### Participant timeline

#### Assessments and visits

The patient will be assessed for change in pain, wound healing, strain during defecation, impression of improvement, and adverse events at 1 week, 6 weeks, and 10 weeks after the start date of the study. For the administration of the questionnaires on pain score, strain during defecation, and patient’s global impression of improvement, the resident or the research assistant (RA) will be trained by the principal investigator to administer it.

During the follow-up visits, the average score for each of the pain score and defecation strain score will be taken for the 3 most recent days that the patient has defecated. The patient will be given a paper dairy to record the pain score and strain score during defecation 3 days prior to the follow-up clinic visit.

### Recruitment

At AUBMC, patients with acute anal fissure are treated at the out-patient department of the hospital by all the general surgeons. The patients treated at the outpatient center will be introduced to the study by their attending physician. If the patient agrees to participate, then the RA will screen the patient according to the defined inclusion and exclusion criteria and if eligible then the RA will discuss the study and obtain his/her informed consent (in private offices within the clinics area). The expected recruitment rate is 4 patients per week.

Patients will not be reimbursed for transportation or time for the first 2 visits to the clinic (at 1 week and 6 weeks) because these are part of clinical care. The last visit to the clinic that will take place 10 weeks will be for follow-up as part of the research, so the patient will be reimbursed for transportation for this visit by the research grant money. The professional fees of the physician for this visit will be also reimbursed by the research grant money, because it is for research purposes.

Participants recruitment was initiated in December 2019 however, due to the impact of both local events and the COVID-19 pandemic no patients have been recruited to the date of this protocol submission. Recruitment phase completion is expected to be extended to December 2021.

### Assignment of interventions

#### Sequence generation

Participants will be randomly assigned to their treatment in a 1:1:1 ratio, according to a computer generated schedule, stratified by type of treatment (MEBO or DTZ or both), using permuted blocks of variable sizes.

#### Allocation Concealment Mechanism & Implementation

Random sequence generation will be selected by CRI biostatistician using computer software. The CRI person will hold details of the blocking and block sizes in a separate document unavailable to those involved in the study including those enrolling patients, collecting data, evaluating outcomes, or analyzing data, so we ensure allocation concealment.

The CRI person will not share the treatment intervention with study personnel until baseline characteristics are collected, and patient is recruited into the trial. The physician will contact the CRI biostatistician and receive treatment allocation, so he or she can prescribe the ointment to which the patient is randomized to.

The allocation will be revealed by the biostatistician to the physician to administer the treatment. However, none of the study team will be informed about the treatment allocation.

#### Blinding (masking)

It will be difficult to blind the physician and patient because of the characteristic smell of MEBO that is easily recognized by the physician or patient. We will blind to allocation the physician who will assess for outcomes, and the research team members who are collecting the data as well as the data analysts.

### Data collection, management, and analysis

#### Data collection methods

Data on basic demographics of the study subjects, relevant risk factors, confounders, and indication for type of treatment will be collected at baseline using protocol specific standardized case report forms, CRF. The CRF will be completed by the research assistant, following informed consent and prior to the administration of treatment. Data will be collected directly from the patient. The lead investigator will train the research assistants to properly fill the CRF.

Evaluators will be requested to fill immediately a CRF each time they perform an outcome evaluation on a study subject. These forms will be handed in the same setting to the research assistant who will take care of storing them.

When a patient fails to comply with the follow up exams, we will collect applicable data on the phone (Appendix C). For those who drop from the study, we will analyze their basic demographic and disease data to determine whether or not they are similar to those who stayed on the study.

#### Data management

The research assistant will enter the data from the CRFs on the study data excel sheet within 1 week of its collection. Clear explanation of all headings and variables in the data collection sheet will be done on a separate word document for future reference. Coding of the data will be clarified from the start on a separate sheet within the same document. Data will be entered as the actual numeric values or the actual categorical variable, initially. In the end, the statistician will code all the data in preparation for analysis. The PI will perform spot checks on the data and will review all the CRF and assessment collection sheets performed within each week.

The principle investigator plans a weekly meeting with the research team to raise and discuss any issues related to data collection, missing data, and retention of patients.

We will have a password security protected laptop for the research assistant. At AUBMC, data will be stored on the AUB intranet server in the PI’s computer. All research members will have access to the data.

The CRFs will be stored in a locked cabinet in the PI’s office 3 years following publication of study results.

We will provide a SOP, standard operating manual, detailing data management procedure to ensure consistency in case of change in the study members.

Audits will be performed monthly on 20% of the charts.

#### Statistical methods

We will compare the trials’ three arms for the primary, secondary, and exploratory outcomes. We will use the chi-square or Fischer exact test if the expected count of any of the outcomes is less than 5 per cell for analysis of incidence of categorical outcomes (like wound healing). We will use independent t-test for analysis of the continuous outcomes (like change in pain score, defecation strain score, Patient’s Global Impression of Improvement). We will calculate relative risk with corresponding 95% confidence intervals to compare incidence of the categorical outcomes and we will report difference in means for the continuous outcomes. SPSS version 20 will be used to conduct the analysis. A 2-sided *p*-value will be set at 5%.

Univariate analysis will be performed separately for the primary and secondary outcomes at 1 week, 6 weeks, and 10 weeks from that start date of the study.

Multivariate analysis for change in pain score and wound healing will be performed using linear and logistic regression respectively, controlling for age, gender, duration of the anal fissure since presentation, analgesic medications intake (only for change in pain score), and defecation strain score.

We will perform both intent-to-treat (ITT) and per-protocol (PP) analysis for all outcome measures. The ITT analysis will include all patients in the arm to which they were randomized. In case of failure of treatment in one of the arms, those who will switch to another arm will be still analyzed in the arm to which they were initially randomized.

Multiple imputation methods will be used to handle missing data. To assess the effect of missing data on the analysis, sensitivity analyses will be performed. The study biostatistician will perform best case scenario, worst case scenario, and group averages. We will assess the baseline characteristics of those who will be lost to follow up, to help us understand what where the potential outcomes.

### Monitoring

#### Data monitoring committee (DMC)

The DMC committee will be independent of the principal investigator and the funders of the trial. It will be composed of a surgeon, an internist, nurse, a biostatistician, and a representative from the patient advocacy office at AUBMC. All members should not have any conflict of interest related to the study. The DMC will be chaired by the internist, who has to keep a record of the meetings and recommendations for future reference. Since this is an investigator-initiated trial, the principal investigator will appoint the DMC members.

The DMC will meet every other month, at times of scheduled interim analysis, and upon conclusion of the study. The primary role of the DMC will be to review accumulating data and alert the steering committee if there are alarming rates of side effects in any arm of the trial. This committee will not have executive power to stop the trial or modify treatment. It will report results to the principal investigator. In addition, the DMC will keep track of accrual rate.

#### Interim analysis

The interim analysis will be done 3 times throughout the study; upon recruiting quarter, two-quarters, and three-quarters of the study population. The study biostatistician who is blind to the treatment allocations will conduct the interim analysis using the O’Brien Fleming stopping rules and will report the results to the DMC confidentially. At each interim-analysis interpretation, the DMC will alert the principal investigator if one arm is found to be beyond doubt either more beneficial or more harmful than the other arm. The PI will take in consideration the results of the interim analysis, opinion of the DMC, and various important factors to decide upon the fate of the trial. The chairperson of the DMC will monitor the clinicaltrials.gov website for registration of new trials and for newly reported results from trials addressing similar question as this trial.

### Efficacy and safety

The adverse effects can be part of the outcome measures we detailed earlier in the protocol or other not specified side effects. Either way, any side effect will be reported, and the subject will be managed according to the standard of care or the preference of the treating physician.

In case any adverse effects arise outside scheduled follow-up time, the patient will be seen by the treating physician in an extra add-on visit. If the treating physician deems it necessary to unblind the research team to treatment allocation then it will be discussed with the PI and DMC who will then convene and decide on unblinding procedure.

In case of failure of the treatment, which is after 6 weeks of treatment, the patient will undergo surgery. The patient may also undergo surgery in case he/she decides to stop the medical therapy during the treatment period and proceed with surgery. There is no standard for the risk of failure of topical therapy to necessitate surgery. Hence, the risk of failure cannot be evaluated with the use of MEBO. The primary physician who is treating the patient will be responsible for the management of the side effects.

### Auditing

The PI will schedule a weekly meeting with all the study members to review eligibility of new participants enrolled in the study, consent forms, all case report forms (CRFs), all assessment sheets filled during that week, adherence to trial interventions and policies, and reports of side effects. The PI will double check the entered data in terms of completeness, timeliness of entry, and correctness of the data. Random checks will also be done.

### Ethics & Dissemination

#### Research ethics approval and consent to participate

We will submit this protocol, informed consent template, case report forms, and other study related appendices to the Institutional Review Board (IRB) at each of the study sites to review the scientific soundness of the project, ethical aspects, and its impact on medical practice for our patient population. The PI will submit progress reports to IRB annually from the date of the first IRB approval and within a month of study completion and later upon termination. No study site other than AUBMC will be part of this study.

After the primary physician introduces the study to the patient, the research assistant will explain the study and invite the patients to sign the consent form. The research assistant will be trained by the principal investigator on the process of signing the consent. In addition, other members of the study team will be trained and certified to obtain the consent, so they can help in case the research assistant was not available to discuss participation with a potential candidate. The subject will be given opportunity to ask questions regarding the study and will receive a copy of the IRB approved and updated consent form (CF) with his/her signature.

#### Protocol amendments

Any modifications to the protocol regarding study objectives, study design, eligibility criteria, sample sizes, or significant changes in the study that will impact study conduct, potential benefit or safety of the study subjects will initially require agreement from the research study data monitoring committee. Then the amendment will be submitted to the IRB for approval before implementation as well as to the trial registry *ClinicalTrials.gov*. The study participants will be notified of study changes and will sign an updated informed consent form reflecting such changes.

#### Confidentiality

All study related forms and information will be stored at the study site, where they are stored in cabinets that can only be accessed by study members. All electronic databases will be password protected. Computers used during this study will also be password protected. Patients’ personal information will be collected only upon recruitment and kept in the trial’s regulatory binder, which will only be accessible via the PI upon valid request.

#### Access to data

The DMC will have access to the data while the study is in progress. At conclusion of the study, principal investigators and co-principal investigators will have full access to the identified data.

#### Dissemination policy

All data and analysis will remain blinded until main outcomes are published. The study results will be communicated to the participants by email, letter by mail, or a phone call by the PI.

We will submit a de-identified dataset to an appropriate data archive after 3 years of study termination to share our data with the surgical community.

#### Authorship

All authors should have contributed to this protocol reasonable and meaningful edits that culminated with this final protocol version. There is no intention to employ any professional writers.

## Supplementary Information

**Additional file 1.** Consent Form English – the consent form used for documenting patient’s consent to participate.

**Additional file 2.** SPIRIT Checklist – the SPIRIT checklist marked for pages relevant to each criteria listed in the SPIRIT guideline.

**Additional file 3.** Cover Letter – letter to the editorial office summarizing the purpose of this protocol and by proxy the trial.

**Additional file 4.** Appendix A – NRS; Numeric Rating Scale for measuring Pain Score associated with the anal fissure as reported by the patient.

**Additional file 5.** Appendix B – Impression of Improvement; Patient’s Global Impression of Improvement for measuring the patient’s take on his/her’s anal fissure improvement.

**Additional file 6.** Appendix C – OTP Questionnaire; Over- the-Phone Questionnaire for collecting certain data from the patient over the phone, in cases where they fail to present to the clinic visit.

**Additional file 7.** Appendix D – SPIRIT Figure; SPIRIT Figure in the shape of a table delineating the participants’ timeline for data collection and clinic visits starting from enrollment and until study completion.

## Data Availability

The data that support the findings of this study are available from the American University of Beirut Medical Center but restrictions apply to the availability of these data, which were used under license for the current study, and so are not publicly available. Data are however available from the authors upon reasonable request and with permission of the American University of Beirut Medical Center.

## Abbreviations

DTZ: Diltiazem
MEBO: Moist Exposure Burns Ointment
AUBMC: American University of Beirut Medical Center
CCB: Calcium Channel Blocker
FDA: Food & Drug Administration
AF: Anal Fissure
NRS: Numeric Rating Scale
GTN: Glyceryl Trinitrate
HIV: Human Immunodeficiency Virus
PI: Principal Investigator
RA: Research Assistant
CRI: Clinical Research Institute
CRF: Case Report Form
SOP: Standard Operating Procedure
SPSS: Statistical Package for Social Sciences
ITT: Intent-to-Treat
PP: Per-protocol
DMC: Data Monitoring Committee
