# Supplementary material for: MEBO versus topical Diltiazem versus a combination of both ointments in the treatment of acute anal fissure: a randomized clinical trial protocol
Source: BMC Complement Med Ther. 2021 Feb 24;21:75. doi: 10.1186/s12906-021-03227-z (PMC7902753; doi:10.1186/s12906-021-03227-z)
Supplement: Supplementary file 1 — Additional file 1. Consent Form English – the consent form used for documenting patient’s consent to participate. [file 12906_2021_3227_MOESM1_ESM.docx]

# Consent to Participate in a Research Study

**Title of Research Study:**

**Comparative evaluation of MEBO Ointment, and Topical Diltiazem ointment in the treatment of acute anal fissure: a Randomized Clinical Trial. (BIO-2018-0547)**

**Investigator: Dr. Eman Sbaity**

Address: American University Hospital

Cairo Street

Beirut, Lebanon

Phone: (01) 350 000 ext 5260

**Site where the study will be conducted:** American University of Beirut Medical Center, Beirut, Lebanon

**You are being asked to participate in a clinical research study conducted at the American University of Beirut. Please take time to read the following information carefully before you decide whether you want to take part in this study or not. Feel free to ask your doctor if you need more information or clarification about what is stated in this form and the study as a whole.**

**PART I: Information Sheet**

**1) Purpose of the research study and overview of participation:**

Anal fissure is a common complication of the anorectal region and one of the most reported causes of anal pain. It can be cured by surgery or medical treatment; however, there is an increase in the use of topical therapy for the treatment of anal fissure to avoid complications post-surgery. One type of topical therapy that is commonly used for anal fissure is Diltiazem, which has been shown to be effective in curing anal fissure. Diltiazem is a calcium-channel blocker that can promote healing of the anal fissure through relaxation of the anal sphincter. Another emerging form of topical therapy for anal fissure is MEBO ointment that is used for the treatment of burns and wound healing. In this study, we aim to compare the efficacy and safety of topical Diltiazem ointment to MEBO ointment in the treatment of anal fissure.

**2)Procedures:**

We expect to recruit an approximate of total 183 participants, with 61 patients in each arm. Your treating physician will approach you and introduce the study during your admission period, before applying the medical therapy. Afterwards, the research assistant will further discuss the study with you.

If you agree to participate in this study, you will be assigned by chance to apply topical Diltiazem ointment only, MEBO ointment only, or a combination of both for a duration of 6 weeks. You are allowed to take stool softeners and pain killers throughout the study. We will collect information about your results at different points in time as follows. We will collect basic information about your health before you receive your medical therapy. The duration of the study will be 2 months and a half. At 1 week, 6 weeks, and 10 weeks from the start date of your therapy, you will visit the clinic to be assessed for wound healing. A member of the research team will also measure your pain using the “0-10 Numeric Pain Rating Scale”. We are also going to use a scale to measure strain during defecation, and the patient’s global impression of improvement. The duration of each visit will be approximately 30 minutes.

**3) Costs:**

The costs of the medication will be covered by the research fund.our 3 clinic visits at 1 week , 6 and 10 weeks from the start date of the study are part of clinical care and will not be covered by the research money. However, your last visit to the clinic that will take place after 10 weeks from the start date of the study will be covered by the research money. The cost of the medications either MEBO or Diltiazem will be paid for from the research money. We will provide you with the medication after you consent to participating in the study. If you fail to attend your clinic visits for evaluation of anal fissure healing, we will contact you by phone to collect applicable data. The phone call will last about 3-5 minutes.

Your participation in this study will not affect you relationship with your treating physician nor with AUBMC.

**4) Risks as a result of participating in the study:**

The anticipated risks when using topical Diltiazem are minor and not frequent , mainly headaches. No risks are anticipated when using MEBO ointment. However, there may be unforeseeable risks from using MEBO or Diltiazem ointment that we are not currently aware of.

The adverse effects can be part of the outcome measures we detailed earlier (pain, wound healing, strain during defecation). Other risks that will be assessed include headache, dizziness, and itching. Either way, any side effect will be reported and the subject will be managed according to the standard of care or the preference of the treating physician.

AUBMC will cover the cost of treating, on its premises, medical adverse events resulting directly from the medication and/or procedures of this research study. Otherwise, it will not cover for the costs of medical care for any medical condition or issue.

**5) Benefits as a result of participating in the study**

If you participate in this study, you will help the physician understand if MEBO ointment cures anal fissure, and if it has better results than Diltiazem.

**6) Any alternative treatment**

In addition to Diltiazem, there are several other topical therapies for anal fissure. Other treatment options for anal fissure include oral medical therapy, injection of botulinum toxin, and surgery. These therapies pose minimal risk to the patient since they are proven to heal anal fissure.

If you agree to participate in this research study, the information will be kept confidential. Unless required by law, only the study doctor and designee, the ethics committee and inspectors from governmental agencies will have direct access to your medical records.

**7) Withdrawal from study after recruitment**

Your decision to enter the study is voluntary and will not affect your care during the period of treatment. You can withdraw from the study at any point with no consequences to your care. If you wish to withdraw at any point please contact the principal investigator Dr. Eman Sbaity at 76/110882 or by email [es25@aub.edu.lb](mailto:es25@aub.edu.lb). The principal investigator may exclude you from the study at any time if you no longer meet the inclusion criteria.

**8) Compensation:**

**You will not be paid for taking part in this study.**

**9) Any significant finding in this study will be conveyed to you. If you want us to contact you at the conclusion of the study, please provide us with your information.**

**PART II: Consent**

**Investigator’s Statement:**

**I have reviewed, in detail, the informed consent document for this research study with (name of patient, legal representative, or parent/guardian) the purpose of the study and its risks and benefits. I have answered to all the patient’s questions clearly. I will inform the participant in case of any changes to the research study.**

**_______________________**

**Name of Investigator or designee Signature**

**Date & Time**

**Patient’s Participation:**

**I have read and understood all aspects of the research study and all my questions have been answered. I understand that refusal to participate will not result in loss of benefits of treatment at the AUB-MC. I voluntarily agree to be a part of this research study and I know that I can contact Dr. Eman Sbaity at 76/110882 or any of his/her designee involved in the study in case of any questions. If I feel that my questions have not been answered, I can contact the Institutional Review Board for human rights at 01-350000 ext 5445. I understand that I am free to withdraw this consent and discontinue participation in this project at any time, even after signing this form, and it will not affect my care or benefits. I understand that the principal investigator may decide to take me off the trial for any reason he considers significant. I know that I will receive a copy of this signed informed consent.**

**__________________________**

**Name of Patient or Legal Representative Signature**

**or Parent/Guardian**

**Date & Time**

**Witness’s Name Witness’s Signature**

**(If patient, representative or parent does not read)**

**Date & Time**
