# Supplementary figures and images for: MEBO versus topical Diltiazem versus a combination of both ointments in the treatment of acute anal fissure: a randomized clinical trial protocol
Source: BMC Complement Med Ther. 2021 Feb 24;21:75. doi: 10.1186/s12906-021-03227-z (PMC7902753; doi:10.1186/s12906-021-03227-z)

**Appendix A: Numerical Rating Scale:**

**
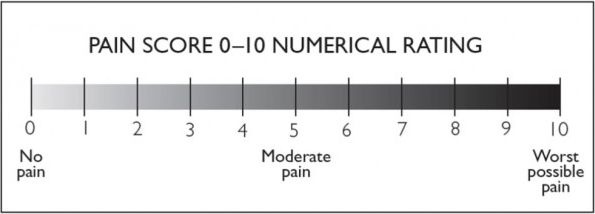
**

Supplement: Supplementary file 4 — Additional file 4. Appendix A – NRS; Numeric Rating Scale for measuring Pain Score associated with the anal fissure as reported by the patient. [file 12906_2021_3227_MOESM4_ESM.docx]

**Appendix B: Patient’s Global Impression of Improvement:**

**
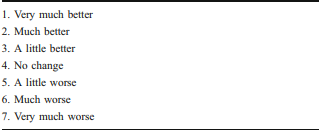
**

Supplement: Supplementary file 5 — Additional file 5. Appendix B – Impression of Improvement; Patient’s Global Impression of Improvement for measuring the patient’s take on his/her’s anal fissure improvement. [file 12906_2021_3227_MOESM5_ESM.docx]
