# Supplementary material for: MEBO versus topical Diltiazem versus a combination of both ointments in the treatment of acute anal fissure: a randomized clinical trial protocol
Source: BMC Complement Med Ther. 2021 Feb 24;21:75. doi: 10.1186/s12906-021-03227-z (PMC7902753; doi:10.1186/s12906-021-03227-z)
Supplement: Supplementary file 6 — Additional file 6. Appendix C – OTP Questionnaire; Over- the-Phone Questionnaire for collecting certain data from the patient over the phone, in cases where they fail to present to the clinic visit. [file 12906_2021_3227_MOESM6_ESM.docx]

**Appendix C:**

**Hello, my name is (name of research assistant or resident physician), I am part of the research team of the study you had participated in at the American University of Beirut Medical Center, for comparative evaluation of the effectiveness of MEBO Ointment and Topical Dilitiazem ointment in the treatment of acute anal fissure: a Randomized Clinical Trial. I am contacting you to collect information for evaluation of healing of anal fissure because you were not able to make it to the clinic visits. I will only collect information that is applicable for data collection over the phone. All answers you give me over the phone are confidential and are for the research purposes of the study. My phone call will last about three to five minutes.**

**Do you have any questions I can answer at this time? (if yes, answer questions; if not, proceed)**

**I would like to ask you the following questions for the evaluation of healing of anal fissure:**

1. **How do you rate your pain on average on a scale from 0-10? Where 0 = no pain and 10 = worst pain imaginable.**

**_____**

1. **How do you rate your strain during defecation on a scale from 0-3?**

**Where 0 = defecation without strain, 1 = mild strain, 2 = moderate strain, and 3 = serious strain.**

**_____**

1. **How do you rate your global impression of improvement on a scale from 1-7?**

**Where 1 = very much better, 2 = much better, 3 = a little better, 4 = no change, 5 = a little worse, 6 = much worse, and 7 = very much worse.**

**_____**

1. **Since the start of your treatment, have you experienced:**
2. **Headaches?**

- **Yes**
- **No**

1. **Itching?**

- **Yes**
- **No**

1. **Dizziness?**

- **Yes**
- **No**

**Thank you for taking your time to complete my questions.**

**Questionnaire to collect applicable data over the phone in Arabic:**

مرحباً ، اسمي (اسم مساعد بحث أو الطبيب المقيم) ، أنا جزء من فريق البحث في الدراسة التي شاركت بها في المركز الطبي في الجامعة الأمريكية في بيروت ، لتقييم مقارن لفعالية مرهم ميبو و دايليتيزم الموضعي في علاج الشق الشرجي الحاد: تجربة سريرية عشوائية. أقوم بالاتصال بك لجمع معلومات لتقييم الشفاء من الشق الشرجي لأنك لم تكن قادراً على الوصول إلى زيارات العيادة. سأجمع فقط المعلومات التي تنطبق على جمع البيانات عبر الهاتف. جميع الإجابات التي تقدمها لي عبر الهاتف سرية وتتم لأغراض البحث الخاصة بالدراسة. سوف تستمر مكالماتي حوالي ثلاثة الى خمسة دقيقة.

هل لديك أي أسئلة يمكنني الإجابة عليها في هذا الوقت؟ (إذا كانت الإجابة بنعم ، أجب عن الأسئلة ، وإذا لم يكن الأمر كذلك ، تابع)

أود أن أطرح عليك الأسئلة التالية لتقييم شفاء الشق الشرجي:

1. كيف تقيم ألمك كمُعَدَّل الـ 3 أيام الماضية ، كيف تقيم ألمك على مقياس من 0 إلى 10؟ حيث 0 = لا يوجد ألم و 10 = أسوأ ألم يمكن تخيله.

_____

2. كيف تقيم إجهاد خلال التغوط على مقياس من 0-3؟

حيث 0 = التغوط بدون إجهاد ، 1 = إجهاد خفيف ، 2 = إجهاد معتدل ، و 3 = إجهاد خطير.

_____

3. كيف تقيم انطباعك العالمي عن التحسن على مقياس من 1-7؟

حيث 1 = أفضل بكثير للغاية ، 2 = أفضل بكثير ، 3 = أفضل قليلاً ، 4 = لا تغيير ، 5 = أسوأ قليلاً ، 6 = أسوأ بكثير ، و 7 = أسوأ بكثير للغاية.

_____

4. منذ بداية العلاج ، هل واجهت:

ا. الصداع؟

- نعم
- لا

ب. الحك؟

- نعم
- لا

ج. دوخة؟

- نعم
- لا

شكرا لأخذ وقتك لإكمال أسئلتي
