# Supplementary material for: MEBO versus topical Diltiazem versus a combination of both ointments in the treatment of acute anal fissure: a randomized clinical trial protocol
Source: BMC Complement Med Ther. 2021 Feb 24;21:75. doi: 10.1186/s12906-021-03227-z (PMC7902753; doi:10.1186/s12906-021-03227-z)
Supplement: Supplementary file 7 — Additional file 7. Appendix D – SPIRIT Figure; SPIRIT Figure in the shape of a table delineating the participants’ timeline for data collection and clinic visits starting from enrollment and until study completion. [file 12906_2021_3227_MOESM7_ESM.docx]

**Appendix D: SPIRIT Figure**

|  | **Study Period** | | | | | |
| --- | --- | --- | --- | --- | --- | --- |
|  | **Enrolment** | **Allocation** | **Post-allocation** | | | **Close-out** |
| **Timepoint** | **Clinic or hospital admission** | **(Day 0)**  **Day of administration of treatment** | **1 week after start date of study** | **6 weeks after start date of study (treatment period)** | **10 weeks after start date of study (follow-up)** |  |
| **Enrollment** | **X** |  |  |  |  |  |
| **Eligibility screen** | **X** |  |  |  |  |  |
| **Informed Consent** | **X** |  |  |  |  |  |
| **Allocation** |  | **x** |  |  |  |  |
| **Interventions** |  | **x** |  |  |  |  |
| MEBO |  | **x** |  |  |  |  |
| DTZ |  | **x** |  |  |  |  |
| **Assessments** |  |  |  |  |  |  |
| Baseline Variables   - Age - Weight - Gender - Average pain score - Average defecation strain score - Medications taken - Laxatives taken - Fiber supplements taken | **X** |  |  |  |  |  |
| **Outcome Variables** |  |  |  |  |  |  |
| Change in Pain score |  |  | **X** | **x** | **x** |  |
| Wound healing |  |  | **X** | **x** | **x** |  |
| Defecation strain score |  |  | **X** | **x** | **x** |  |
| Patients’ global impression of improvement |  |  | **X** | **x** | **x** |  |
| **Adverse events** |  |  |  |  |  |  |
| Headache |  |  | **X** | **x** | **x** |  |
| Itching |  |  | **X** | **x** | **x** |  |
| Dizziness |  |  | **X** | **x** | **x** |  |
| Vital signs and sensitivity reactions |  |  | **X** | **x** | **x** |  |
