# Supplementary material for: MEBO versus topical Diltiazem versus a combination of both ointments in the treatment of acute anal fissure: a randomized clinical trial protocol
Source: BMC Complement Med Ther. 2021 Feb 24;21:75. doi: 10.1186/s12906-021-03227-z (PMC7902753; doi:10.1186/s12906-021-03227-z)
Supplement: Supplementary file 3 — Additional file 3. Cover Letter – letter to the editorial office summarizing the purpose of this protocol and by proxy the trial. [file 12906_2021_3227_MOESM3_ESM.docx]

Manuscript title: **MEBO versus topical Diltiazem versus a combination of both ointments in the treatment of acute anal fissure: a randomized clinical trial protocol**

Article Type: Protocol for Randomized Controlled Trial

Anal fissure is a common complaint that crosses gender, age and ethnic barriers. It can cause severe pain and discomfort that may render daily activities requiring a sitting position very difficult to tolerate. Many agents have been utilized for the treatment of anal fissures with some proving superior to others. Topical Diltiazem is one such commonly used medication to relieve and cure anal fissures. MEBO ointment is a medication known in Eastern countries for its burn and wound healing properties. We propose that the MEBO ointment alone or in combination with Diltiazem ointment, could be superior to topical Diltiazem in treating the pain and healing the wound associated with acute anal fissures.

Declaration: Neither this manuscript nor any part of it was published before or is currently being considered for publication in any other journal. Further statements of declarations are provided in a section titled Declarations.

Responses to Formatting Changes requirements by BMC Complementary Medicine and Therapies:

1. Ethical and funding approval documentations were emailed as scanned copies to [BMCSeriesEditorial@biomedcentral.com](mailto:BMCSeriesEditorial@biomedcentral.com)
2. The study protocol has undergone peer-review by the funding body mentioned in the Declarations section of the submission.
3. The study is currently recruiting patients and has not yet completed the recruitment process i.e. study status is at ongoing recruitment.
4. This study has no results yet as it is still in the recruitment phase. As such there are no submissions or publications of any results for this study.
